# Foliar Sprays of Multi-Nutrient Fertilizer Containing Selenium Produce Functional Tomato Fruits with Higher Shelf Life

**DOI:** 10.3390/plants13162288

**Published:** 2024-08-17

**Authors:** Everton Geraldo de Morais, Maila Adriely Silva, Anyela Pierina Vega Quispe, Gilson Gustavo Lucinda Machado, Debora Teixeira Prado, Pedro Antônio Namorato Benevenute, Jucelino de Sousa Lima, Gustavo Ferreira de Sousa, Eduardo Valério de Barros Vilas Boas, Luiz Roberto Guimarães Guilherme

**Affiliations:** 1 Department of Soil Science, Federal University of Lavras, University Campus, P.O. Box 3037, Lavras 37203-202, Minas Gerais, Brazil; 2 Department of Food Science, Federal University of Lavras, University Campus, P.O. Box 3037, Lavras 37203-202, Minas Gerais, Brazil; gilsonguluma@gmail.com (G.G.L.M.);; 3 Department of Biology, Institute of Natural Sciences, Federal University of Lavras, University Campus, P.O. Box 3037, Lavras 37203-202, Minas Gerais, Brazil

**Keywords:** food waste, biofortification, firmness

## Abstract

Selenium (Se) is a nutrient whose daily intake is often below the recommended levels in people. Biofortification with Se is a method to increase this intake by raising the Se concentration in tomato fruits, an effect dependent on sources and modes of application. Additionally, Se application can promote the enhancement of other compounds in tomato fruits, altering their metabolism, which may increase the fruit’s shelf life. This study aimed to determine how different strategies of applying a multi-nutrient fertilizer containing Se (SeMNF) can increase the Se content and other bioactive compounds and enhance the shelf life of tomato (*Solanum lycopersicum* L.) fruits. Different foliar fertilization strategies involving the use of SeMNF were evaluated in field trials conducted on commercial tomato crops. Indeterminate-growth tomatoes were used, and different Se doses and application strategies were tested. Harvesting was conducted in three phases according to fruit ripening. Each harvested fruit was assessed for the Se content, macro and micronutrients, total phenolic compounds, vitamin C, antioxidant activity, carotenoids, pH, total titratable acidity, and total soluble solids in tomato fruits. Doses of 15 g ha^−1^ of Se, split into three applications, increased the Se content in the fruits at 1 and 2 harvests. The application of SeMNF at Se doses above 10 g of Se ha^−1^ increased firmness, days of ripening, and the nutritional quality of the tomatoes (higher contents of carotenoids (+39%), lycopene (+33%), antioxidant activity (+16%), total phenolic compounds (+38%), and vitamin C (+14%) in a dose-dependent effect of the application strategy used. These results contributed to an increase in the shelf life of tomatoes, consequently reducing food waste.

## 1. Introduction

Selenium (Se) is an essential human micronutrient involving various physiological and metabolic processes [1,2]. This element is associated with enhancing the human immune system, and its average intake recommendation for adults is 60–70 μg day^−1^ [2,3]. However, its consumption in the human diet is generally low, and worldwide, approximately one billion people are Se deficient [4]. It occurs due to a low concentration of Se in foods, an effect related to the low natural availability of this element in the soil, which consequently limits its accumulation by plants [3,5]. In addition to the low Se intake, the availability and beneficial effects of Se in the human body against diseases are highly dependent on the chemical form in which Se is found. Organic forms derived from plant products are more efficient in preventing certain diseases than inorganic forms of Se commonly used in medications [6].

Among strategies to increase the Se concentration in edible parts, genetic and agronomic biofortification are more used, with agronomic biofortification being more efficient for Se [3]. Agronomic biofortification involves Se application through fertilization, aiming for more significant accumulation in the edible part of the plant [3]. Several studies in different countries indicate and justify the efficiency and environmental safety of this method of nutritional enrichment with Se, using various crops for this purpose [7,8,9]. Agronomic biofortification depends on various interacting factors such as the application method, doses, application timing, sources used, soil type, and plant species [2,3,5,8,9,10], with foliar application more efficient than soil fertilization [9]. The primary sources of Se used in agricultural foliar spray are salts like sodium selenate and sodium selenide. However, recent studies showed that using multi-nutrient fertilizer with Se may increase Se uptake/redistribution by plants compared with selenium salts [7].

The ability to accumulate Se depends on the plant species and their interaction with different factors, and biofortification programs target those species with more significant nutrient accumulation potential [2,3,5]. The tomato plays a central role in global nutrition, being consumed worldwide [10,11,12]. Additionally, it contains a variety of biologically active compounds such as carotenoids (including lycopene), proteins, minerals, dietary fiber, and oils and is rich in antioxidant and anticarcinogenic compounds, including polyphenols [10,11,12,13]. Moreover, when tomato is consumed in the human diet, it exhibits antioxidant and antimutagenic activities and protects against oxidative stress [10,11,12,13]. Despite the existence of the literature indicating that tomato is classified as a non-accumulator plant of Se, which would hinder its inclusion in Se-biofortified food production programs [3], recent studies demonstrate the potential of tomato in agronomic biofortification programs [14,15,16]. Selenium biofortification of tomato plants is highly dependent on the stage of development at which Se is applied, with promising results happening when the application is made starting from the beginning of flowering, yet further studies are needed in this matter, especially for indeterminate growth plants [17,18].

In addition to increasing Se levels in tomato fruits, Se application promotes the improvement of the nutritional quality of the tomato plant, increasing firmness, phenolic compound levels, antioxidant activity, and carotenoid contents such as lycopene [14,15,16]. Furthermore, Se can act on the metabolic pathway of ethylene synthesis [15]. These factors together increase the shelf life of tomatoes, contributing to reducing losses from harvest to final consumption (food waste) [14,15,16].

In addition, Se can act as a mitigator of different types of climate-related stresses and diseases, reducing losses until the harvesting stage (food loss) [8,19,20,21]. Approximately 54% of the tomato production is estimated to be lost [22]. Food loss contributes to the increase in greenhouse gas emissions [23], and globally, food loss and waste account for 8% of anthropogenic greenhouse gas emissions, with a significant portion coming from primary production. Thus, Se application emerges as a promising technique for biofortification and a sustainable strategy for reducing losses. However, for a biofortification process to be efficient, it is necessary to test different biofortification strategies in commercial production systems that ensure both the maintenance and/or increase in the yield and the food’s nutritional quality. Some Se applications can increase the content in edible parts, but with a reduction in the plant yield [14].

In this context, our study hypothesized that applying multi-nutrient fertilizer containing Se (SeMNF) using different strategies promotes biofortification and the improvement of the nutritional quality of the tomato, extending its shelf life. Therefore, this study’s aims were: (i) to assess the effect of different strategies of SeMNF application on tomato plant production; (ii) to investigate how SeMNF application can increase the Se content in tomato fruits, as well as their nutritional quality, evaluating different compounds in tomato fruits; (iii) to determine if SeMNF application can increase the shelf life of tomato fruits; and (iv) to determine how potential improvements in nutritional quality due to the increase in bioactive compounds in tomato fruits can enhance their shelf life. Based on an extensive literature review, we believe this is the first time Se biofortification in tomatoes has been evaluated under these perspectives.

## 2. Results

### 2.1. Leaf Analysis

There were no significant differences in the N, P, K, Ca, Mg, S, Fe, Cu, Mn, Zn, and B content in the leaves according to the treatments tested (*p* > 0.05) (Table A1). Compared with the control (T1—without Se application), only T4 and T5 increased the Se content in the leaves (an average increase of 352% in both treatments) (Table 1). In the fresh leaf samples, there were no significant differences in the malondialdehyde (MDA) and activities of superoxide dismutase (SOD), catalase (CAT), and ascorbate peroxidase (APX) (*p* > 0.05) (Table 1). The content of H_2_O_2_ was reduced by treatments T4 and T5, with an average reduction of 50% compared with the control (T1). Peroxidase activity (POD) increased by T5 (+70%) and T2 (+55%), and it was reduced by T4 (−52%) when compared with the control (Table 1). 

### 2.2. Tomato Fruits

There was no significant difference (*p* > 0.05) among the treatments in terms of the tomato yield and production according to the height and diameter (Table 2). When summing the three harvests throughout the cultivation period, the average yield in the experimental area was 82 t ha^−1^. The height and diameter of the fruit, the number of fruits per plant (FN), and the average fruit mass (AFM) (Table 2) were also not affected by the studied treatments (*p* > 0.05). 

**Figure 1:**
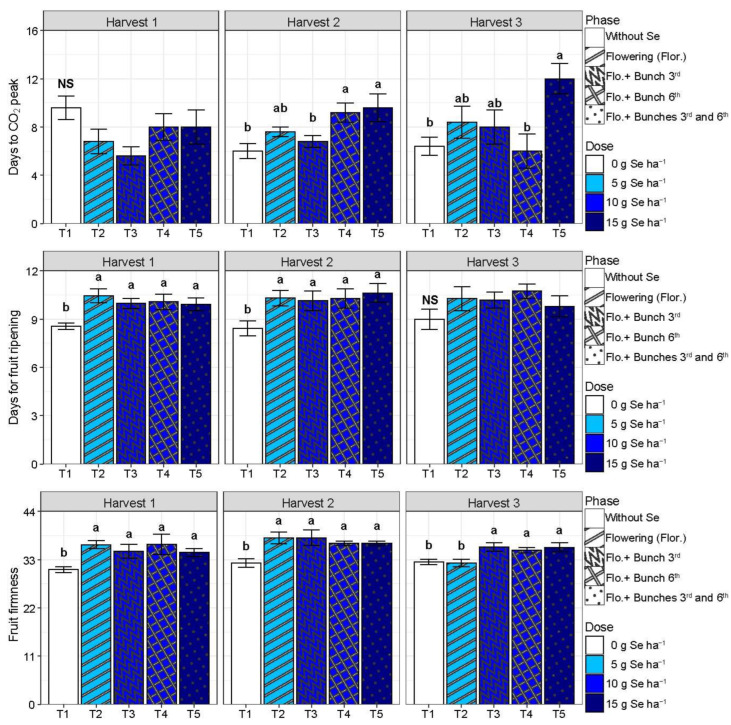
Days to CO_2_ peak, time of ripening, and fruit firmness according to different strategies of multi-nutrient fertilizer (MNF) application containing selenium in each harvest evaluated. T1: Control (without application of MNF); T2: Application at the beginning of flowering (2 kg ha^−1^ of MNF providing 5 g of Se ha^−1^); T3: Application at the beginning of flowering +3rd bunch (4 kg ha^−1^ of MNF providing 10 g of Se ha^−1^); T4: Application at the beginning of flowering +6th bunch (4 kg ha^−1^ of MNF providing 10 g of Se ha^−1^); T5: Application at the beginning of flowering +3rd bunch +6th bunch (6 kg ha^−1^ of MNF providing 15 g of Se ha^−1^). NS: statistically non-significant (*p* > 0.05). Bars with standard error of each treatment followed by the same letter in each column are not differentiated by the Duncan test (*p* > 0.05) in each harvest evaluated.

There was no difference between the treatments studied on color variables for the tomatoes evaluated at the red ripeness stage (*p* > 0.05) (Table A2). Selenium did not affect the timing of the climacteric peak of tomatoes from the first harvest (*p* > 0.05). On the other hand, the highest dose of Se (T5) delayed it in tomatoes from the second and the third harvest, while Se at the second highest dose (T4) did it just in the second harvest (*p* < 0.05) (Figure 1). Compared with T1, in harvest 2, T4 and T5 delayed the climacteric peak by about 3.4 days, and in harvest 3, T5 delayed it by around 5.6 days (Figure 1).

Despite the treatment, Se retarded the ripening, based on the changing of the peel color from green to red, of fruit from the first and second harvest for 1.55 to 1.93 days (*p* < 0.05), although no effect has been noted in fruit from the third harvest (*p* > 0.05) (Figure 1). Fruits from all treatments involving the SeMNF application (T2, T3, T4, and T5) were, on average, 16% firmer than the control fruit (T1) in harvests 1 and 2 (Figure 1). However, fruit from harvest 3 presented firmness 10% higher than the control, just when the Se dose applied was ≥10 g ha^−1^ (T3, T4, and T5) (Figure 1).

**Figure 2:**
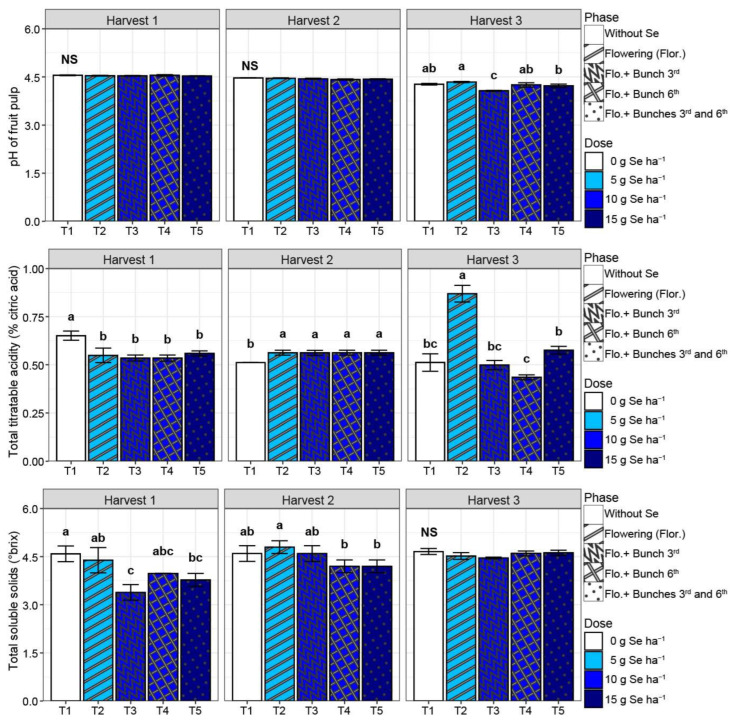
pH, total titratable acidity, and total soluble solids in fruit pulp according to different strategies of multi-nutrient fertilizer (MNF) application containing selenium in each harvest evaluated. T1: Control (without application of MNF); T2: Application at the beginning of flowering (2 kg ha^−1^ of MNF providing 5 g of Se ha^−1^); T3: Application at the beginning of flowering +3rd bunch (4 kg ha^−1^ of MNF providing 10 g of Se ha^−1^); T4: Application at the beginning of flowering +6th bunch (4 kg ha^−1^ of MNF providing 10 g of Se ha^−1^); T5: Application at the beginning of flowering +3rd bunch +6th bunch (6 kg ha^−1^ of MNF providing 15 g of Se ha^−1^). NS: statistically non-significant (*p* > 0.05). Bars with standard error of each treatment followed by the same letter in each column are not differentiated by the Duncan test (*p* > 0.05) in each harvest evaluated.

**Figure A1:**
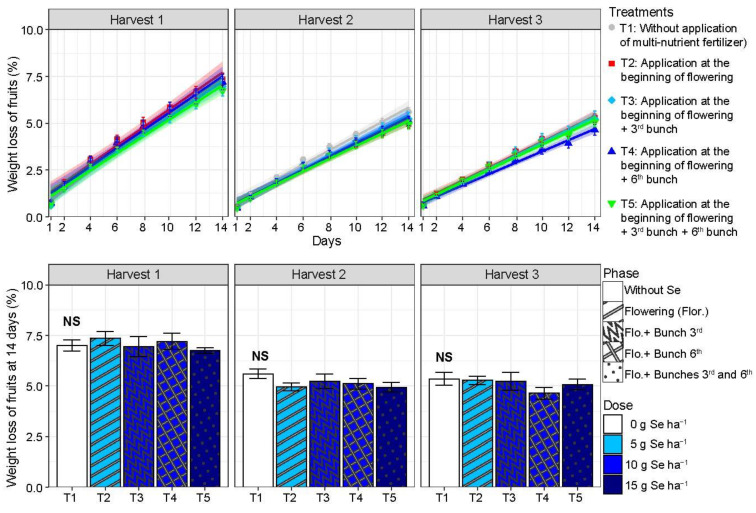
Weight loss of fruits according to different strategies of multi-nutrient fertilizer application containing selenium in each harvest evaluated. T1: Control (without application of multi-nutrient fertilizer); T2: Application at the beginning of flowering (2 kg ha^−1^ of multi-nutrient fertilizer providing 5 g of Se ha^−1^); T3: Application at the beginning of flowering +3rd bunch (4 kg ha^−1^ of multi-nutrient fertilizer providing 10 g of Se ha^−1^); T4: Application at the beginning of flowering +6th bunch (4 kg ha^−1^ of multi-nutrient fertilizer providing 10 g of Se ha^−1^); T5: Application at the beginning of flowering +3rd bunch +6th bunch (6 kg ha^−1^ of multi-nutrient fertilizer providing 15 g of Se ha^−1^). NS: statistically non-significant (*p* > 0.05).

The mass loss in tomato fruits did not differ between the treatments studied despite harvest (*p* > 0.05). Fruit from harvests 1, 2, and 3 presented a mass loss of 7.5%, 5.1%, and 5.1%, respectively, after 14 days of ripening (Figure A1). In the fruit pulp, the pH was affected by treatments only in harvest 3, and T3 reduced the pH (−5%) compared with T1 (Figure 2). The total titratable acidity reduced (−17%) in harvest 1 and increased (+8%) in harvest 2 for all treatments involving the SeMNF application (T2, T3, T4, and T5) compared with T1 (Figure 2). In harvest 3, only the lower rate of Se applied (T2) increased the TTA by 70% compared with T1. Compared with T1, the total soluble solids (TSS) were affected only by T3 and T5 in harvest 1, with a reduction of 22% (Figure 2).

**Figure 3:**
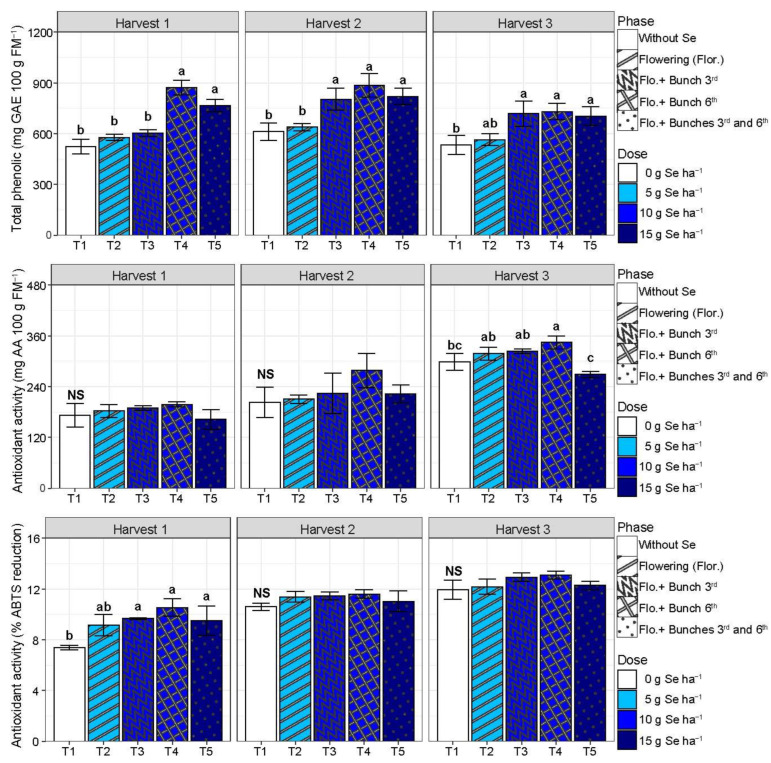
Total phenolic content and antioxidant activity by the phosphomolybdenum complex (mg of ascorbic acid -AA) and ABTS reduction methods according to different strategies of multi-nutrient fertilizer (MNF) application containing selenium in each harvest evaluated. T1: Control (without application of MNF); T2: Application at the beginning of flowering (2 kg ha^−1^ of MNF providing 5 g of Se ha^−1^); T3: Application at the beginning of flowering +3rd bunch (4 kg ha^−1^ of MNF providing 10 g of Se ha^−1^); T4: Application at the beginning of flowering +6th bunch (4 kg ha^−1^ of MNF providing 10 g of Se ha^−1^); T5: Application at the beginning of flowering +3rd bunch +6th bunch (6 kg ha^−1^ of MNF providing 15 g of Se ha^−1^). NS: statistically non-significant (*p* > 0.05). Bars with standard error of each treatment followed by the same letter in each column are not differentiated by the Duncan test (*p* > 0.05) in each harvest evaluated.

The total phenolic content increased by 56% in harvest 1 for T4 and T5 compared with T1 (Figure 3). In harvests 2 and 3, T3, T4, and T5 increased the total phenolic content by an average of 36 and 34% compared with T1, respectively, to harvests 2 and 3. The antioxidant activity determined by the phosphomolybdenum complex did not differ between treatments in harvests 1 and 2. However, in harvest 3, only T4 increased (+15%) compared with T1 (Figure 3). The antioxidant activity determined by the ABTS reduction method increased (+7%) by T3, T4, and T5 compared with T1, and in harvests 2 and 3, the treatments did not differ between them (*p* > 0.05) (Figure 3).

**Figure 4:**
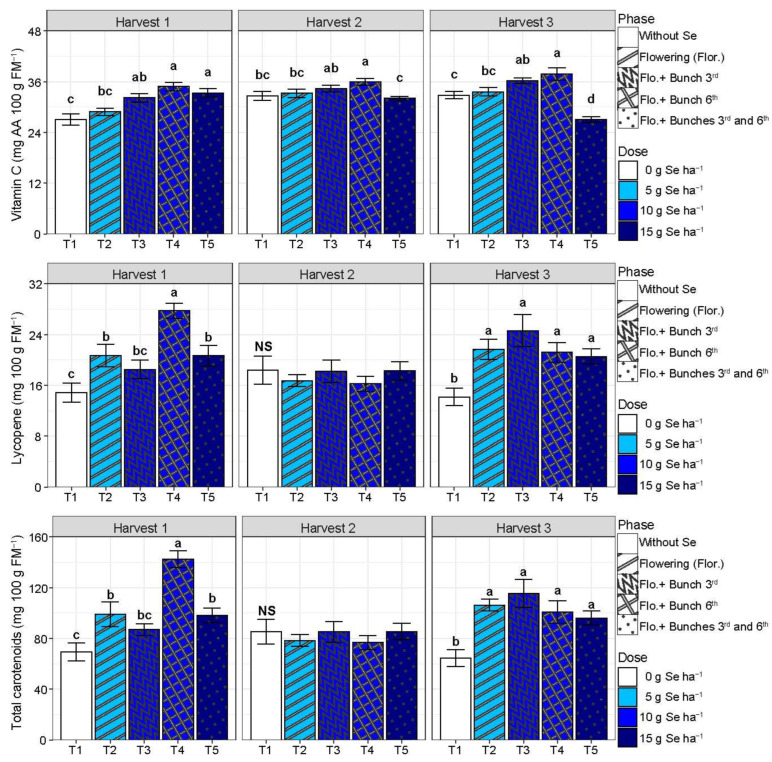
Vitamin C (mg of ascorbic acid—AA), lycopene, and total carotenoids’ evaluation according to different strategies of multi-nutrient fertilizer (MNF) application containing selenium in each harvest. T1: Control (without application of MNF); T2: Application at the beginning of flowering (2 kg ha^−1^ of MNF providing 5 g of Se ha^−1^); T3: Application at the beginning of flowering +3rd bunch (4 kg ha^−1^ of MNF providing 10 g of Se ha^−1^); T4: Application at the beginning of flowering +6th bunch (4 kg ha^−1^ of MNF providing 10 g of Se ha^−1^); T5: Application at the beginning of flowering +3rd bunch +6th bunch (6 kg ha^−1^ of MNF providing 15 g of Se ha^−1^). NS: statistically non-significant (*p* > 0.05). Bars with standard error of each treatment followed by the same letter in each column are not differentiated by the Duncan test (*p* > 0.05) in each harvest evaluated.

Treatments T3, T4, and T5 promoted higher levels of vitamin C (+24%) in fruit from harvest 1, compared with T1, while just T4 did it in fruit from harvest 2 (+10%) and T3 and T4 (+13%) in fruit form harvest 3 (*p* < 0.05). In addition, the control fruit from harvest 3 presented a higher level of vitamin C (+18%) than the T5 fruit (*p* < 0.05) (Figure 4). Lycopene and total carotenoids increased with T2, T4, and T5 compared with T1 in harvest 1, with higher values with T5, showing increments of 87% and 105%, respectively, for lycopene and total carotenoids (Figure 4). In harvest 2, there was no difference between treatments for lycopene and total carotenoids (*p* > 0.05). In harvest 3, all treatments involving the SeMNF application (T2, T3, T4, and T5) increased lycopene by 55% and total carotenoids by 63% compared with T1.

**Figure 5:**
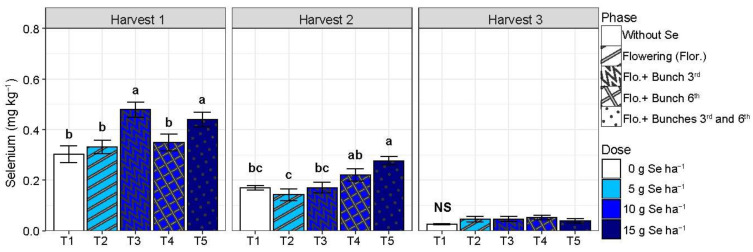
Determination of selenium (Se) content in dried tomato fruits according to different strategies of multi-nutrient fertilizer (MNF) application containing Se in each harvest evaluated. T1: Control (without application of MNF); T2: Application at the beginning of flowering (2 kg ha^−1^ of MNF providing 5 g of Se ha^−1^); T3: Application at the beginning of flowering +3rd bunch (4 kg ha^−1^ of MNF providing 10 g of Se ha^−1^); T4: Application at the beginning of flowering +6th bunch (4 kg ha^−1^ of MNF providing 10 g of Se ha^−1^); T5: Application at the beginning of flowering +3rd bunch +6th bunch (6 kg ha^−1^ of MNF providing 15 g of Se ha^−1^). NS: statistically non-significant (*p* > 0.05). Bars with standard error of each treatment followed by the same letter in each column are not differentiated by the Duncan test (*p* > 0.05) in each harvest evaluated.

In harvest 1, only Cu was affected by the treatments, and T3 and T4 increased by 45% compared with T1 (Table 3). In harvest 2, the treatments affected only nitrogen (N) and boron (B). The nitrogen content in the fruit increased by 32% in T5 compared with T1, and both T2 and T5 increased B in the fruit by 31% compared with T1. In harvest 3, the treatments affected only zinc (Zn) in the fruit, with T4 and T5 increasing Zn by 79% compared with T1. Selenium in the fruit increased by 52% in T3 and T5 compared with T1 in harvest 1, enhancing the Se content to 0.46 mg kg^−1^ (Figure 5). In harvest 2, only T5 increased the Se in the fruit compared with T1, with an increment of 63%, enhancing the Se content to 0.28 mg kg^−1^. In harvest 3, there was no difference between the treatments studied for Se in the fruit (*p* > 0.05), with mean values of 0.04 mg kg^−1^.

### 2.3. Principal Component Analysis 

**Figure 6:**
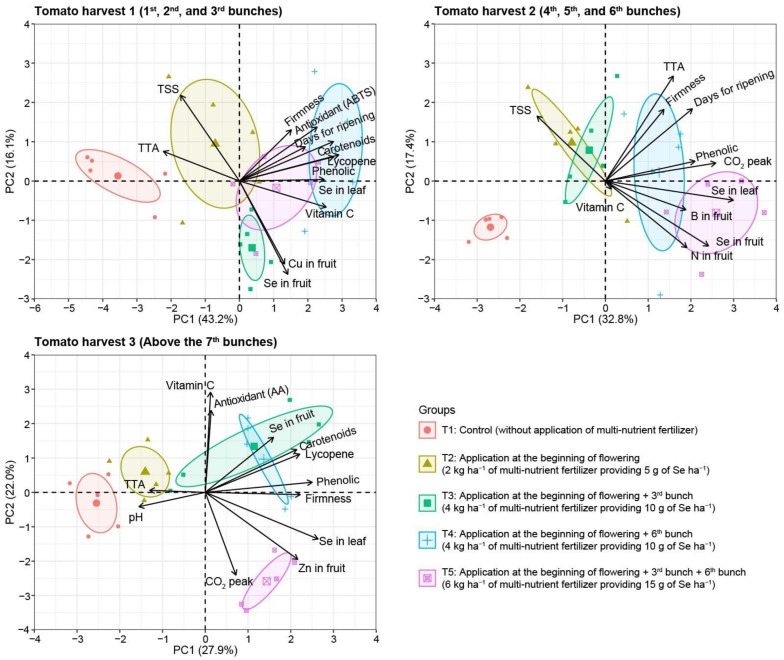
Principal component analysis of each harvest evaluated. pH: pH in fruit pulp; TTA: total titratable acidity in fruit pulp; TSS: total soluble solids in fruit pulp; CO_2_ peak: days to CO_2_ peak, Ripening: days for fruit ripening; Firmness: fruit firmness; Vitamin C: Vitamin C—mg of ascorbic acid in fruit pulp; Lycopene: Lycopene content in fruit pulp; Carotenoids: total carotenoids content in fruit pulp; Phenolic: total phenolic content in fruit pulp; Antioxidant (AA): antioxidant activity in fruit pulp by the phosphomolybdenum complex methods (mg of ascorbic acid); Antioxidant (ABTS): antioxidant activity in fruit pulp by ABTS reduction method; Zn in fruit, Cu in fruit, B in fruit, N in fruit, and Se in fruit: concentration of zinc, copper, boron, nitrogen, and selenium (Se) in dried tomato fruits, respectively; Se in leaf: concentration of Se in dried tomato leaves; PC: principal component.

In harvest 1, the fruit firmness, antioxidant activity (ABTS reduction method), time of ripening, total carotenoid content, lycopene content, total phenolic content, and vitamin C content had a positive relationship among them, also related to a higher content of Se in leaves favored by T4 and T5 and a negative relation with the control treatment (T1) (Figure 6). In harvest 2, the Se in the leaf, Se in fruit, N in fruit, B in fruit, days to CO_2_ peak, total phenolic content, time of ripening, and firmness had a positive relationship among them favored by T4 and T5 and a negative relationship with T1. In harvests 1 and 2, TSS had a negative relationship with Se in fruit. In harvest 3, Se in the fruit and leaf had a positive relationship with the total carotenoid content, lycopene content, total phenolic content, and fruit firmness favored by T3 and T4 and a negative relation with T1 and T2, and treatment with the lowest rate of Se applied (5 g ha^−1^). Additionally, Se in the fruit and leaf in harvest 3 had a negative relation with the TTA and pH in the fruit pulp.

## 3. Discussion

Selenium is not a plant nutrient, according to the current criteria adopted in mineral plant nutrition [24,25]. However, many studies have shown that Se improved crop growth and production when plants are cultivated under different abiotic and biotic stresses [8,19,20,26]. The increase in crops production through Se application, mainly occurring in stress conditions, involves the ability of Se to act on various metabolic processes and enzymes such as CAT, SOD, and APX, reducing the amount of ROS like H_2_O_2_ and consequently reducing the lipid peroxidation [8,19,27,28]. Several types of abiotic stresses commonly observed in field conditions, such as high light intensity, high temperature, water deficit, and low nutrient availability, reduce the photosynthetic rate of plants, consequently diminishing the tomato yield [29]. However, although our study has shown that the application of SeMNF increased the foliar Se content and reduced the amount of H_2_O_2_, this effect did not enhance tomato production or influence the antioxidant system (SOD, CAT, or APX) as well as the lipid peroxidation (MDA) of the fresh leaves (Table 1). In our study, tomato cultivation conditions involved the use of fertigation + shading net + high soil fertility, which were adequate conditions justifying the absence of a response to the use of SeMNF in tomato plants once the application of Se also did not affect the total tomato yield, as well as the yield in different diameter classes (Table 2).

The tomato production from the experimental area was 82.0 t ha^−1^, a yield considered high compared to the Brazilian average of 69.1 t ha^−1^ [30]. Additionally, there was a predominance of fruits with a diameter greater than 60 mm (classified as large), a diameter class used for the commercial classification of salad-type fruits, as used in the experiment. Usually, larger-diameter tomatoes have greater acceptance in the consumer market [31]. In addition to the effect of Se by itself, the application of SeMNF promotes the input of other nutrients that may affect crop production nutrition. However, there was no change in the levels of macro- and micronutrients in the tomato leaves and concerning tomato production (Table A1). Our results contrast with Silva [7], where applying SeMNF (a different nutrient proportion compared to our study) increased soybean production, with a direct relationship between production and the N absorbed by soybeans.

Despite not obtaining productivity gains due to the use of SeMNF, there was an improvement in the post-harvest quality of tomato fruits (Figure 1, Figure 2, Figure 3 and Figure 4). In Brazil, it is estimated that from harvest to the final consumer, approximately 54% of the tomato production is lost [22]. Thus, practices that extend the shelf life are essential for sustainable agriculture. Here, we observed that the SeMNF application increased the shelf life by evaluating different variables, such as the increase in the time of ripening and fruit firmness in all SeMNF application strategies in harvests 1 and 2. In addition, the increased fruit firmness in harvest 3 was observed for strategies that applied a Se amount ≥ 10 g ha ha^−1^. In a meta-analysis performed by Xu et al. [16], the authors did not find the effect of Se on tomato fruit firmness. Still, the authors highlighted that the impact depends on an interaction of factors (tomato varieties and Se fertilizer concentrations). In line with our study, tomato cultivation under greenhouse conditions showed that a foliar spray of 1 mg Se L^−1^ as sodium selenate improved fruit firmness starting at 10 days of storage, an effect related to the ability of Se to stimulate the activity of antioxidant enzymes and non-enzymatic antioxidants [32], similarly to what was observed in our study (Figure 6).

Tomatoes are climacteric fruits that present an increase in their ethylene production and respiration rate during ripening. The earlier the climacteric starts, the shorter the shelf life of the fruit. At the highest dose (T5), Se delayed the climacteric CO_2_ peak of tomatoes from harvest 2 and 3, whereas the second highest dose (T4) delayed it in tomatoes just from harvest 2 (Figure 1). Although Se did not significantly affected the respiratory climacteric in fruit of the first harvest, it extended the time of ripening of those fruit (Figure 1). Selenium also extended the time of ripening of fruit from the second harvest, despite the dose, although only T4 and T5 have retarded the climacteric peak (Figure 1). On the other hand, the retardant effect of T5 on the climacteric peak of fruit from the third harvest did not promote an extension on the time of ripening of those fruits (Figure 1).

The observed effect of Se in increasing the time of ripening due to the application of SeMNF has already been verified for plants treated with Se. It includes several mechanisms, such as ROS inhibition by stimulating antioxidant defense systems [15]. Additionally, the increase in the fruit ripening time by Se may promote the downregulation of ethylene biosynthesis genes [15,28]. This effect occurs because Se can be incorporated into the amino acid methionine [33], and methionine is an amino acid precursor in ethylene synthesis in plants, a hormone responsible for fruit ripening [28,34,35]. In apples, Badalar [28] observed that after harvest, evaluating a storage period of 6 months, the foliar application of Se reduced ethylene production compared to no application of Se. This reduction in ethylene production can be indirectly verified by the increase in the number of days to the peak of carbon dioxide (CO_2_) because the final reaction of ethylene involving its oxidation generates CO_2_ [36]. This effect was observed due to the application of SeMNF, using 15 g of Se combined with an application in three phases (beginning of flowering +3rd bunch +6th bunches), increasing the number of days to the peak of CO_2_ (Figure 1).

Recent studies have demonstrated the beneficial effects of Se biofortification on the shelf life of various types of fruits. For instance, the application of a nutrient solution containing 8 mg L^−1^ of Se every 15 days reduced the mass loss from 12.3% (without Se) to 7.5% (Se application), consequently increasing fruit firmness correlated with the increase in the total phenolic content, antioxidant activity, and lycopene levels [14]. In addition, applying Se nanoparticles on strawberry plants significantly reduced mass loss, respiration rate, and ascorbic acid degradation during refrigerated storage [37]. It also occurs because Se modulates the gene expression related to carbohydrate and lipid metabolism in fruits. For example, in strawberries, Se biofortification increased the expression of the genes involved in ascorbic acid (vitamin C) synthesis and lipid degradation. This increase may contribute to maintaining fruit quality during storage, thereby improving overall quality [16,32]. These findings are corroborated by our study, where the application of Se increased the antioxidant activity, total phenolic content, vitamin C, carotenoids, TTA, TSS, and pH. These factors directly correlate with the enhancement of fruit firmness, the number of days until flowering, and the peak CO_2_ levels of the fruits (Figure 6). Similarly to our study, applying Se via a foliar spray increased the vitamin C content in tomato fruits [38]. Additionally, a meta-analysis on Se application in tomatoes showed that Se increases vitamin C levels and enhances TSS, TTA, and the lycopene content, resulting in tomatoes richer in bioactive compounds [16].

It was also possible to observe a relationship between the Se content in the leaf and fruit and the total carotenoid content (Figure 6). The correlation between carotenoids and Se is related to its incorporation into amino acids such as methionine and cysteine, forming selenomethionine and selenocysteine [19]. These amino acids (cysteine and methionine) are precursors of isopentenyl pyrophosphate and dimethylallyl pyrophosphate, which are precursors of all carotenoids [39]. Thus, Se application may increase carotenoid levels, as observed in our study and other studies with tomatoes [14,16] and carrots [40]. Furthermore, the antagonistic relationship between the fruit pH and TTA and the increase in the shelf life in harvests 2 and 3 due to SeMNF application is related to the fact that the acidity of tomato fruits can activate enzymes involved in the synthesis of volatile compounds such as ethylene, which would eventually stimulate ripening and fruit deterioration [14,28]. Thus, it was possible to observe that several mechanisms acted together to increase tomato fruits’ nutritional quality and shelf life.

Despite the application of Se from multi-nutrient fertilizer being a promising technique, both in increasing the quality of the tomato or production, as well as the Se content in edible parts [7], its efficiency was highly dependent on the quantity and frequency of the application (Figure 1, Figure 2, Figure 3, Figure 4, Figure 5 and Figure 6). Selenium used in foliar sprays is preferably absorbed by the leaf and then translocated to the reproductive organs [3,41]. Foliar sprays at different stages are essential for Se applied in early stages or in crops with indeterminate growth, where flowers, fruits at intermediate ripening stages, and ripe fruits suitable for harvest can all be present on the same plant. Thus, it may occur that applications in early stages, depending on the quantity of SeMNF, only affect the first harvests (T2), and strategies involving application at multiple stages tend to be more effective in producing tomatoes with a longer shelf life and quality (T3, T4, and T5), a fact observed in our study.

Tomato plants are considered plants with a low Se accumulation capacity [3]. In addition, applying lower Se quantities may be ineffective in increasing the Se content in the harvested fruit, as observed in our study. Here, the increase in Se levels in the first and second harvests was maximized by the application of 15 g Se ha, divided into flowering +3 bunch +6 bunch, allowing for achieving fruit content values of 0.46 mg kg^−1^ in harvest 1 and 0.28 mg kg^−1^ in harvest 2 (Figure 5). In a field study, the foliar application of Se at the fruit development stage of the first and second inflorescences using 150 g ha^−1^ was able to achieve Se levels in dry matter higher than in our study, reaching values of 0.99 mg kg^−1^ [42]. However, the authors did not report whether there was any effect on tomato production. Based on the average tomato consumption per day (55 g of tomato per day) [43] and the fruit moisture mean of 94,7% [44], the fruits of treatment 5 in harvest 1 and 2 provided 1.90 and 1.16 µ of Se per day, respectively, equivalent to less than 4% of the adequate intake of Se in humans (50 to 70 µ of Se day^−1^) [3]. In this way, it is observed that the strategies adopted effectively increased the levels of Se in tomato fruits. Moreover, they significantly enhanced the produced tomatoes’ nutritional quality and shelf life.

## 4. Materials and Methods

### 4.1. Treatments and Experimental Conditions

**Figure 7:**
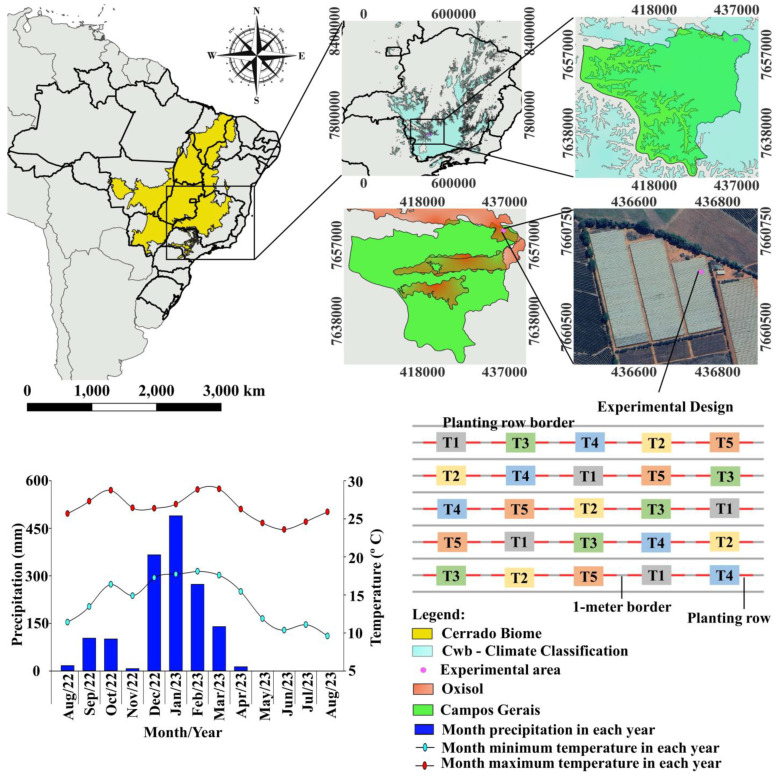
Location in UTM zones, design, and climatic data during the experiment.

The experiment was carried out with tomatoes (*Solanum lycopersicum* L.) of an Italian type under commercial field conditions in the years 2022–2023 in an area located in the city of Campos Gerais, Minas Gerais, Brazil (21°09′15.8″ S; 45°36′32.8″ W), to validate different Se application strategies. The local climate is classified as Cwb according to Köppen [45], characterized by a humid temperate climate with a dry winter and moderately hot summer and an average annual temperature of 16.5 and 25.4 °C, minimum and maximum, respectively. The rainy season occurs mainly between October and March, with an average yearly precipitation of 121.25 mm. According to the Brazilian soil classification system, the soil at the study site was classified as Latossolo Vermelho [46], related to the 0–0.20 m layer, as shown in Table 4, which corresponds to Ferralsols [47] and Oxisols in the Soil Taxonomy [48], adopted as the official classification in the study (Figure 7).

All fertilization in the soil during tomato growth was provided through a fertirrigation system. The experiment was a randomized complete block design, with five treatments and five replicates with a 1.5 × 0.55 m spacing between lines and plants, respectively. The treatments included foliar application of SeMNF, using different strategies, and the Se doses were established in previous experiments. The SeMNF used contained 10.0, 26.2, 149.4, 30, 2.5, 2.5, and 5 g kg^−1^ of N, P, K, Mg, Se, Zn, and B, respectively. The treatments were applied at different phenological stages, as described in Table 5. The experimental plots consisted of a line measuring 7 m long. Each block consisted of a planting row. Between blocks, a planting row was established to prevent drift effects during the application of treatments (Figure 7). Between treatments in the same planting row (block), a 1 m border was designated to avoid the effect, as mentioned above. Treatment application was carried out using a pressurized backpack sprayer with CO_2_. Each experimental plot was individually sprayed with 400 mL of the spray solution containing the tested treatments. In the control treatment, which involved the absence of SeMNF application, only distilled water was applied.

Seven days after the last treatment described in Table 5 was sprayed, leaves from the middle third of the tomato plants were collected. These leaves were dried in a forced air circulation oven at 45 °C until constant to determine Se, macro-, and micronutrient contents. Additionally, at the same plant position mentioned in the above collection, expanded leaves were collected and immediately covered with plastic bags and aluminum foil, submerged in liquid nitrogen, and stored in an ultra-freezer at −80 °C until biochemical analyses. The biochemical analyses performed included the determination of malondialdehyde (MDA) and hydrogen peroxide (H_2_O_2_) contents and activities of superoxide dismutase (SOD), ascorbate peroxidase (APX), catalase (CAT), and peroxidase (POD).

The fruit harvest was performed at the commercial harvest point (tomato fruits ranging from green to pink color) in three stages, harvesting fruits from three plants in each central row. In the first stage (harvest 1), tomatoes from the lower third of the plant (1st, 2nd, and 3rd bunches) were harvested. In the second stage (harvest 2), fruits from the middle third (4th, 5th, and 6th bunches) were collected, and in the last stage (harvest 3), fruits from the upper third of the plant (above the 7th bunches) were harvested.

### 4.2. Biochemical Analysis in the Leaves

In the leaves collected and stored in the ultra-freezer, 0.20 g of fresh mass was macerated in a mortar with liquid nitrogen and homogenized in 1500 µL of trichloroacetic acid (TCA) 0.1%, and centrifuged at 12,000× *g* for 15 min at 4 °C. In the extracts, the H_2_O_2_ content (nmol g^−1^ of fresh mass) was determined through a reaction with potassium iodide (KI) and readings performed in a spectrophotometer at 390 nm [50]. To assess lipid peroxidation in the same extract, the malondialdehyde (MDA) content was determined by quantifying substances reactive to 2-thiobarbituric acid (TBA) according to the methodology described by Heath and Parker [51]. The reaction of TBA (2-thiobarbituric acid) with the formation of final lipid peroxidation products was used to quantify MDA. The readings for MDA content (nmol g^−1^ of fresh mass) were taken in a spectrophotometer at 535 and 600 nm, and values were calculated according to Heath and Packer [51].

For the determination of enzymatic activity, 0.20 g of fresh mass was macerated in liquid nitrogen with the subsequent addition of 1.5 mL of a buffered solution (0.1 mol L^−1^ of potassium phosphate pH (7.8), 0.1 mol L^−1^ of EDTA (pH 7.0), 0.5 mol L^−1^ of DTT, 0.1 mol L^−1^ PMSF, 1 mmol L^−1^ of ascorbic acid, and 22 mg of PVPP). The suspension obtained was centrifuged at 13,000× *g* for 10 min at 4 °C, and then the supernatant was collected. In the supernatant, the activity of superoxide dismutase (SOD) was determined according to the methodology described by Beauchamp and Fridovich [52]. Aliquots of supernatant were transferred to light-protected test tubes containing 50 mmol L^−1^ of potassium phosphate buffer at pH 7.8, including 0.1 mmol L^−1^ of EDTA, 13 mmol L^−1^ of L-methionine, and 750 µmol L^−1^ of nitroblue tetrazolium (NBT). The reaction was initiated by adding 2 mmol L^−1^ riboflavin, and the tubes were quickly transferred to a chamber illuminated by a 30-watt lamp (30 µmol photons m^−2^ s^−1^) for 7 min. The reaction was stopped by turning off the light, and readings were taken at 560 nm. Enzyme activity was estimated based on NBT inhibition, and one unit of activity was the amount of enzyme required to inhibit 50% of its reduction, expressed as U SOD min^−1^ g^−1^ of fresh mass.

The activity of ascorbate peroxidase (APX) was determined following the method described by Nakano and Asada [53]. Aliquots of the supernatant were added to a reaction medium of 2.7 mL of 50 mmol L^−1^ potassium phosphate buffer (pH 6.0) containing 0.5 mmol L^−1^ of L-ascorbic acid. The reaction was initiated by adding 0.2 mL of 30 mmol L^−1^ of H_2_O_2_ and monitored by the decrease in absorbance at 290 nm using a spectrophotometer over 120 s, with successive readings at 30 s intervals. The APX activity was estimated using the 2.8 mmol L^−1^ cm^−1^ molar extinction coefficient for ascorbate at 290 nm and expressed as nmol ASA g^−1^ min^−1^ of fresh mass. 

For peroxidase activity (POD) determination, the POD reaction solution contained 100 µL of 30 mmol L^−1^ H_2_O_2_, 100 µL of guaiacol, and 100 μL of enzymatic extract (supernatant) in 2.7 mL of sodium phosphate buffer. The absorbance of POD samples was observed in a time scan (0–60 s) at 470 and 240 nm, respectively, using a spectrophotometer, and the results were expressed in nmol Tetraguaiacol g^−1^ min^−1^ of fresh mass [54]. 

Catalase (CAT) activity was performed as described by Azevedo et al. [55], based on H_2_O_2_ consumption. The reaction medium consisted of 100 mmol L^−1^ of potassium phosphate buffer (pH 7.0), 12.5 m mol L^−1^ of H_2_O_2_, water, and enzymatic extract (supernatant). Activity was monitored by the decrease in absorbance at 240 nm for two minutes and incubated at 28 °C. Results were expressed as nmol H_2_O_2_ min^−1^ g^−1^ of fresh mass.

### 4.3. Post-Harvest Analysis in Tomato Fruits

After fruit harvesting, the number of fruits per plant and the fruit diameter and height were determined. Fruit mass was also measured to assess productivity. The harvested fruits were stored at 20 °C until the red ripening stage when fruit quality analyses were conducted. The samples of fruits at the red ripening stage were macerated, creating a paste that was spread onto a tray to a thickness of about 5 cm, and then placed in a forced air circulation oven at 45 °C until constant for subsequent determination of Se, macro-, and micronutrient contents.

Representative fruits from each treatment at the break stage were separated and weighed on days 1, 2, 4, 6, 8, 10, 12, and 14 to assess weight loss. In these same fruits, fruit respiration was also measured by placing them in a closed glass chamber for one hour, and after this time, the CO_2_ (%) in the chamber was determined using a portable CO_2_ meter (PBI Dansensor, Check point, Tendric Pacific, Saffron Walden, England). The number of days to reach the peak of CO_2_ was determined based on this information.

Six representative fruits per plot were separated from each experimental unit at the break-ripening stage. The color change of these fruits was monitored daily. When these fruits reached the red ripening stage, the number of days to define the ripening period was noted, followed by determining the fruit color and firmness. Fruit firmness was measured by penetrating the fruit pericarp with a Marconi benchtop digital penetrometer, model MA 933, equipped with a 2 mm diameter cone tip at a constant speed of 0.01 m min^−1^. Fruit color was assessed using a CR-400 colorimeter (Konica Minolta Sensing Americas, Inc., Ramsey, NJ, USA). After these analyses, the seeds were removed and the pulp was frozen for further analysis.

### 4.4. Fruit Quality

In the tomato pulp samples, the following variables were determined at a ratio of 1 g of pulp to 3 mL of distilled water: pH, total titratable acidity (TTA), and total soluble solids content (TSS). The pH was determined using a pH meter (Tecnal^®^, Piracicaba, Brazil). Total titratable acidity was determined via titration using NaOH 0.1 M, and results were expressed in mg of citric acid 100 g^−1^ of fresh sample. The TSS was determined using an ATAGO PR-100 digital refractometer (Tokyo, Japan). Extracts were initially obtained to determine total phenolic compounds and antioxidant activity, and this procedure was adapted from Rufino et al. [56]. One gram of the sample was weighed into a centrifuge tube, followed by adding 4 mL of 50% methanol and 4 mL of 70% acetone. The mixture was homogenized and shaken on a shaker table for 30 min without light. Subsequently, the tubes were ultrasonicated for 30 min, followed by filtration. The filtrate was transferred to a volumetric flask, and the volume was adjusted to 10 mL. Then, it was stored in amber bottles in the freezer.

In the obtained extract, total phenolic compounds were determined using the Fast Blue method, as described by Medina [57], with some adaptations. An aliquot of 50 µL of the extract was mixed with 200 µL of distilled water, 25 µL of Fast Blue reagent (0.1%, *v*/*v*), and 25 µL of sodium hydroxide (5%, *w*/*v*), and the absorbance was measured at 420 nm after 1.5 h of incubation in the dark. The results were reported as gallic acid equivalents in mg per 100 g of fresh sample mass (mg GAE 100 g^−1^ fresh mass).

In the obtained extract, antioxidant activity was determined using the phosphomolybdenum complex method, where the determination of phosphomolybdenum is based on the reduction of Mo^6+^ to Mo^5+^. The analysis was carried out as described by Prieto et al. [58] with some adaptations. Initially, 50 µL of the sample extract +450 µL of distilled water and 1.5 mL of the phosphomolybdenum complex were pipetted into screw-capped tubes, which were closed, shaken, heated in a water bath at 95 °C for 90 min, cooled in an ice bath, and read on a spectrophotometer at 695 nm. The above process was carried out to determine antioxidant activity using vitamin C to construct the curve. The results were then expressed in mg of ascorbic acid per 100 g sample. 

The antioxidant activity was also determined using the ABTS method, which measures the activity based on the scavenging of (ABTS^+^). The (ABTS^+^) solution was prepared by reacting 2,2’-azinobis (3-ethylbenzothiazoline-6-sulfonic acid) diammonium salt at a concentration of 7 mmol L^−1^ with potassium persulfate at 2.45 mmol L^−1^ at room temperature for 16 h. The solution was then diluted with ethanol to an absorbance of 0.70 ± 0.05 at 734 nm. Aliquots of 5 μL of sample extracts were pipetted into each well of a flat-bottomed 96-well microplate, and 295 μL of (ABTS^+^) solution was added to each well. After 6 min of reaction time in the dark, the absorbance was measured at 734 nm using the same microplate reader mentioned earlier. The results of antioxidant activity were expressed as % ABTS Reduction, according to Auzanneau et al. [59].

To determine vitamin C, 5 g of the fresh pulp mass was homogenized in 45 mL of 0.5% oxalic acid using a Polytron. Subsequently, the homogenate was placed on a shaking table for 30 min and filtered. The extract was then used to determine the ascorbic acid content after the oxidation of ascorbic acid and dehydroascorbic acid by the colorimetric method using 2,4-dinitrophenylhydrazine, as described by Strohecker et al. [60]. The results were expressed in mg of ascorbic acid per 100 g^−1^ fresh mass.

To determine lycopene and total carotenoid contents, 5.0 g of the fresh pulp sample were added to 40 mL of acetone, and the samples were then shaken for 1 h using a circular shaker. Subsequently, filtration was carried out using a funnel wrapped in aluminum foil to prevent photo-oxidation of the pigments. Each sample was washed with acetone three more times to ensure the complete extraction of pigments. Afterward, 45 mL of petroleum ether was added to the separation funnel. The pigments were then transferred, in small fractions, along with distilled water, to the separation funnel, discarding the lower phase. Following this, the samples were washed four times to remove all acetone. Reading on the spectrophotometer and other quantification procedures were performed according to Rodriguez-Amaya [61] by measuring absorbance at 444, 450, 456, 462, and 470 nm. The results were expressed in mg of lycopene or total carotenoids 100 g^−1^ g of fresh mass.

### 4.5. Selenium and Nutrients Contents in Leave and Fruits

In the dried leaf and fruit samples from each harvest, analyses of the products were carried out according to international Quality Assurance/Quality Control (QA/QC) protocols and utilizing certified reference materials (CRMs). The samples and CRMs were analyzed using standard protocols (e.g., digestion using the USEPA 3051A method, followed by ICP-MS analysis). To determine Se, macro-, and micronutrient contents, the samples were dried in a forced-air circulation oven at 45 °C and ground (<0.38 mm) using a stainless steel mill.

Sample digestion was carried out using a microwave oven with a CEM^®^ Mars-5 microwave system (CEM Corp, Matthews, NC, USA), following the USEPA Method 3051A [62]. Selenium concentration in the digested solution was quantified by inductively coupled plasma mass spectrometry (ICP-MS). In contrast, the P, K, Ca, Mg, S, Fe, Mn, Cu, Zn, and B levels were quantified by inductively coupled plasma optical emission spectrometry (ICP-OES). The N content in the dried samples was determined by sulfuric acid digestion, followed by distillation and titration using the Kjeldahl method [63]. During the analyses, certified samples of plant material were employed in quality control and assurance protocols (QA/QC), with a Se recovery rate of 109 ± 9.0%. Tomato leaves—NIST SRM 1573a and White clover—BCR 402 were used as CRMs.

### 4.6. Statistical Analysis

All statistical analyses were carried out using the R software version 4.3.1 [64] with the stats, agricolae, corrplot, factoextra, FactoMineR, and Metrics R packages [64,65,66,67,68]. Treatment means of variables measured in leaf, and each harvest was compared using the Duncan test (*p* < 0.05) after meeting the basic assumptions of analysis of variance (normality, homoscedasticity, additivity, and independence of residuals) and reaching significance of the F-test (*p* < 0.05). A principal component analysis (PCA) was performed to evaluate the relationship between variables. The PCA was performed for each harvest, selecting the contents of Se in the leaf and fruit and the variables that showed significant differences in univariate analyses.

## 5. Conclusions

Applying multi-nutrient fertilizer containing Se did not affect tomato plant production or its morphological parameters. However, regardless of the strategy used, its application increased the number of days for fruit ripening and fruit firmness in the three analyzed harvests. The application of multi-nutrient fertilizer at Se doses above 10 g of Se ha^−1^ increased the nutritional quality of tomatoes, raising the contents of carotenoids, lycopene, the antioxidant activity, total phenolic compounds, and vitamin C in a dose-dependent effect of the application strategy used. The highest Se dose (15 g of Se^−1^) had the highest Se content in tomato fruits. The application of Se, leading to these improvements, contributed to the simultaneous enhancement of nutritional quality and shelf life in tomatoes, proving an efficient and promising strategy to reduce food waste (losses from harvest to consumer). However, further research should be conducted to find approaches to increase Se levels in the final harvests of indeterminate tomato varieties, as the strategies used did not succeed in increasing Se levels in the most recent harvest.

## Figures and Tables

**Table 1:** Selenium (Se) content in dried leaves and contents of malondialdehyde (MDA) and hydrogen peroxide (H_2_O_2_) and activities of superoxide dismutase (SOD), catalase (CAT), ascorbate peroxidase (APX), and peroxidase (POD) in fresh leaves according to different strategies of multi-nutrient fertilizer application containing Se.

Treatment	Se (mg kg^−1^)	MDA (nmol MDA g^−1^ of FM)	H_2_O_2_ (nmol H_2_O_2_ g^−1^ of FM)	SOD (U SOD min^−1^ g^−1^ of FM)	CAT (nmol H_2_O_2_ min^−1^ mg^−1^ of FM)	APX (nmol ASA g^−1^ of FM min^−1^)	POD (nmol Tetraguaiacol g of FM ^−1^ min^−1^)
T1	2.4 ± 0.3 b	17.5 ± 1.4	804 ± 100 a	166 ± 2	909 ± 80	1014 ± 54	2090 ± 330 c
T2	2.9 ± 0.4 b	13.4 ± 1.6	829 ± 121 a	163 ± 1	905 ± 34	998 ± 92	3231 ± 352 b
T3	4.8 ± 0.5 b	16.4 ± 1.8	558 ± 88 ab	160 ± 2	864 ± 49	1173 ± 48	1939 ± 488 c
T4	10.2 ± 0.5 a	18.2 ± 1.3	464 ± 106 b	159 ± 2	812 ± 55	979 ± 20	1001 ± 377 d
T5	11.5 ± 0.5 a	16.6 ± 1.8	343 ± 86 b	159 ± 2	966 ± 60	1053 ± 36	3554 ± 287 a
*p*-value	0.001	0.344 **^NS^**	0.014 *	0.140 **^NS^**	0.152 **^NS^**	0.553 **^NS^**	0.003 **^NS^**

FM: fresh mass. T1: Control (without application of multi-nutrient fertilizer (MNF)); T2: Application at the beginning of flowering (2 kg ha^−1^ of MNF providing 5 g of Se ha^−1^); T3: Application at the beginning of flowering +3rd bunch (4 kg ha^−1^ of MNF providing 10 g of Se ha^−1^); T4: Application at the beginning of flowering +6th bunch (4 kg ha^−1^ of MNF providing 10 g of Se ha^−1^); T5: Application at the beginning of flowering +3rd bunch +6th bunch (6 kg ha^−1^ of MNF providing 15 g of Se ha^−1^). **^NS^**: statistically non-significant (*p* > 0.05); *: statistically significant (*p* < 0.05). Means with standard error within each treatment group that shares the same letter in each column are not considered statistically different according to the Duncan test (*p* > 0.05).

**Table 2:** Total productivity (Total), productivity divided by each transverse diameter class (>60 mm; >50 mm and <60 mm; >40 mm and <50 mm; <40 mm), and non-commercial standard productivity (Non-Com,), height and diameter of the fruit, number of fruits per plant (FN), and average fruit mass (AFM) according to different strategies of multi-nutrient fertilizer application containing selenium in each harvest.

**Tomato Harvest 1 (1st, 2nd, and 3rd Bunches)**										
**Treatment**	**Yield (t ha**^−1^)						**Height (mm)**	**Diameter (mm)**	**FN**	**AFW (g fruit** ^−1^ **)**
	**Total**	**>60 mm**	**>50 and <60 mm**	**>40 and <50 mm**	**<40 mm**	**Non-Com.**				
T1	26.8 ± 1.2	23.7 ± 1.7	2.7 ± 1.1	0.2 ± 0.1	0.1 ± 0.1	0.0 ± 0.0	65.9 ± 1.1	51.3 ± 0.9	21.6 ± 1.3	102.8 ± 3.7
T2	26.1 ± 1.1	23.3 ± 1.6	2.2 ± 0.5	0.3 ± 0.1	0.1 ± 0.1	0.2 ± 0.1	66.2 ± 1.4	51.5 ± 0.7	20.6 ± 0.9	104.9 ± 4.9
T3	26.3 ± 0.5	23.0 ± 0.5	2.8 ± 0.5	0.4 ± 0.1	0.0 ± 0.0	0.1 ± 0.1	66.3 ± 0.3	51.1 ± 0.6	21.5 ± 0.5	101.3 ± 2.1
T4	26.5 ± 1.1	22.1 ± 1.4	3.6 ± 0.1	0.7 ± 0.2	0.1 ± 0.0	0.0 ± 0.0	63.5 ± 1.2	49.6 ± 0.7	23.3 ± 0.4	94.1 ± 3.9
T5	26.3 ± 1.5	22.3 ± 1.9	3.3 ± 0.4	0.4 ± 0.1	0.1 ± 0.1	0.2 ± 0.1	65.1 ± 1.6	50.8 ± 0.9	22.1 ± 0.9	98.3 ± 4.8
*p*-value	0.997 **^NS^**	0.954 **^NS^**	0.435 **^NS^**	0.217 **^NS^**	0.366 **^NS^**	0.564 **^NS^**	0.553 **^NS^**	0.640 **^NS^**	0.572 **^NS^**	0.598 **^NS^**
**Treatment**	**Yield (t ha**^−1^)						**Height (mm)**	**Diameter (mm)**	**FN**	**AFM (g fruit** ^−1^ **)**
	**Total**	**>60 mm**	**>50 and <60 mm**	**>40 and <50 mm**	**<40 mm**	**Non-Com.**				
T1	24.1 ± 1.6	20.9 ± 1.9	2.4 ± 0.4	0.6 ± 0.1	0.2 ± 0.1	0.1 ± 0.1	62.7 ± 1.3	52.1 ± 1.1	20.7 ± 0.8	95.8 ± 5.1
T2	24.2 ± 0.9	19.8 ± 1.3	3.3 ± 0.4	0.5 ± 0.3	0.2 ± 0.1	0.4 ± 0.3	62.8 ± 0.8	51.3 ± 0.9	21.8 ± 0.8	91.7 ± 3.9
T3	24.3 ± 0.9	21.0 ± 0.6	2.5 ± 0.4	0.5 ± 0.1	0.1 ± 0.1	0.3 ± 0.2	63.3 ± 1.0	52.0 ± 0.8	21.6 ± 1.0	93.1 ± 3.7
T4	23.2 ± 0.9	18.5 ± 1.2	3.4 ± 0.5	0.9 ± 0.2	0.2 ± 0.1	0.2 ± 0.1	61.7 ± 0.6	50.2 ± 0.5	22.4 ± 0.4	85.5 ± 2.7
T5	26.5 ± 1.1	22.9 ± 1.3	2.6 ± 0.5	0.6 ± 0.1	0.3 ± 0.1	0.2 ± 0.1	62.7 ± 0.8	51.3 ± 0.5	24.1 ± 0.9	91.0 ± 2.5
*p*-value	0.361 **^NS^**	0.221 **^NS^**	0.312 **^NS^**	0.535 **^NS^**	0.688 **^NS^**	0.844 **^NS^**	0.924 **^NS^**	0.654 **^NS^**	0.206 **^NS^**	0.576 **^NS^**
**Treatment**	**Yield (t ha**^−1^)						**Height (mm)**	**Diameter (mm)**	**FN**	**AFM (g fruit** ^−1^ **)**
	**Total**	**>60 mm**	**>50 and <60 mm**	**>40 and <50 mm**	**<40 mm**	**Non-Com.**				
T1	29.9 ± 0.9	23 ± 1.8	5.1 ± 1.2	1.5 ± 0.7	0.3 ± 0.1	0.0 ± 0.0	60.3 ± 1.6	50.3 ± 1.3	26.3 ± 1.9	95.2 ± 6.5
T2	32.7 ± 1.7	27.4 ± 2.2	4.2 ± 0.7	0.9 ± 0.3	0.2 ± 0.1	0.0 ± 0.0	61.3 ± 0.7	50.6 ± 0.5	28.3 ± 1.2	95.4 ± 2.2
T3	30.3 ± 1.5	24.4 ± 0.7	4.3 ± 0.7	1.3 ± 0.6	0.2 ± 0.1	0.1 ± 0.1	60.7 ± 1.5	48.2 ± 0.7	28.1 ± 2.5	90.3 ± 3.4
T4	31.3 ± 1.4	25.8 ± 1.2	4.8 ± 0.7	0.5 ± 0.2	0.2 ± 0.1	0.0 ± 0.0	61.7 ± 1.1	50.4 ± 1.1	26.8 ± 2.1	97.4 ± 4.5
T5	30.9 ± 1.1	26.6 ± 1.2	3.6 ± 0.7	0.7 ± 0.2	0.1 ± 0.0	0.0 ± 0.0	61.6 ± 0.4	49.5 ± 0.6	26.5 ± 1.0	96.4 ± 2.3
*p*-value	0.776 **^NS^**	0.400 **^NS^**	0.654 **^NS^**	0.700 **^NS^**	0.721 **^NS^**	0.587 **^NS^**	0.885 **^NS^**	0.369 **^NS^**	0.944 **^NS^**	0.785 **^NS^**

T1: Control (without application of multi-nutrient fertilizer (MNF)); T2: Application at the beginning of flowering (2 kg ha^−1^ of MNF providing 5 g of Se ha^−1^); T3: Application at the beginning of flowering +3rd bunch (4 kg ha^−1^ of MNF providing 10 g of Se ha^−1^); T4: Application at the beginning of flowering +6th bunch (4 kg ha^−1^ of MNF providing 10 g of Se ha^−1^); T5: Application at the beginning of flowering +3rd bunch +6th bunch (6 kg ha^−1^ of MNF providing 15 g of Se ha^−1^). **^NS^**: statistically non-significant (*p* > 0.05).

**Table 3:** Content of macro- and micronutrients in dried tomato fruits varied according to different strategies of multi-nutrient fertilizer application containing selenium, assessed at each harvest.

**Tomato Harvest 1 (1st, 2nd, and 3rd Bunches)**											
**Treatment**	**N**	**P**	**K**	**Ca**	**Mg**	**S**	**Fe**	**Cu**	**Mn**	**Zn**	**B**
	^______________________________^ **(g kg^−1^)** ^________________________________^						^_____________________________^ **(mg kg^−1^)** ^_____________________________^				
T1	34.2 ± 1.3	5.8 ± 0.4	33.4 ± 6.6	2.5 ± 0.3	2.5 ± 0.3	2.7 ± 0.3	92.5 ± 6.8	31.6 ± 1.1 **b**	21.9 ± 0.9	36.5 ± 4.6	18.4 ± 3.2
T2	33.0 ± 1.2	6.2 ± 0.8	58.1 ± 5.4	2.8 ± 0.3	2.5 ± 0.3	2.9 ± 0.4	77.2 ± 8.8	33.3 ± 4.6 **b**	22.8 ± 2.0	37.3 ± 4.7	19.1 ± 2.4
T3	34.1 ± 1.6	5.9 ± 0.4	42.5 ± 9.4	2.6 ± 0.1	2.6 ± 0.1	2.8 ± 0.1	103.1 ± 4.6	48.3 ± 2.2 **a**	23.1 ± 1.3	44.0 ± 2.3	18.4 ± 0.7
T4	34.0 ± 1.3	6.7 ± 0.5	38.6 ± 10.5	2.7 ± 0.4	2.7 ± 0.3	3.1 ± 0.3	83.1 ± 8.6	43.2 ± 5.0 **a**	21.2 ± 1.3	42.6 ± 4.9	19.8 ± 2.3
T5	32.2 ± 1.3	6.6 ± 0.3	42.6 ± 7.9	2.8 ± 0.4	2.6 ± 0.1	3.1 ± 0.2	97.3 ± 7.1	32.0 ± 2.0 **b**	21.7 ± 0.7	39.4 ± 2.3	20.1 ± 1.2
*p*-value	0.530 ^NS^	0.647 ^NS^	0.367 ^NS^	0.966 ^NS^	0.923 ^NS^	0.900 ^NS^	0.160 ^NS^	0.001 *	0.788 ^NS^	0.555 ^NS^	0.967 ^NS^
**Treatment**	**N**	**P**	**K**	**Ca**	**Mg**	**S**	**Fe**	**Cu**	**Mn**	**Zn**	**B**
	^______________________________^ **(g kg^−1^)** ^________________________________^						^_____________________________^ **(mg kg^−1^)** ^_____________________________^				
T1	21.7 ± 0.5 **b**	3.5 ± 0.2	41.6 ± 1.6	1.7 ± 0.1	2.1 ± 0.1	2.1 ± 0.1	64.7 ± 2.4	16.0 ± 1.2	15.6 ± 0.5	23.3 ± 0.9	13.0 ± 0.7 **b**
T2	23.1 ± 1.8 **b**	4.1 ± 0.2	47.7 ± 1.2	1.9 ± 0.1	2.5 ± 0.1	2.3 ± 0.1	63.9 ± 1.7	16.5 ± 2.7	19.4 ± 1.4	26.3 ± 1.8	16.6 ± 0.9 **a**
T3	22.8 ± 1.0 **b**	3.4 ± 0.2	44.3 ± 1.3	1.7 ± 0.1	2.3 ± 0.1	2.2 ± 0.1	57.6 ± 2.2	12.7 ± 1.2	16.2 ± 0.5	22.7 ± 0.8	14.8 ± 0.3 **ab**
T4	21.8 ± 1.5 **b**	3.8 ± 0.3	42.8 ± 2.8	1.8 ± 0.1	2.3 ± 0.3	2.3 ± 0.3	58.0 ± 4.1	13.1 ± 2.0	16.7 ± 1.3	24.6 ± 2.9	14.9 ± 1.3 **ab**
T5	28.7 ± 1.3 **a**	4.2 ± 0.3	46.6 ± 2.8	2.1 ± 0.1	2.5 ± 0.2	2.5 ± 0.1	64.6 ± 1.2	13.9 ± 1.9	18.2 ± 0.9	26.7 ± 2.5	17.6 ± 1.0 **a**
*p*-value	0.015 *	0.125 ^NS^	0.260 ^NS^	0.073 ^NS^	0.257 ^NS^	0.488 ^NS^	0.154 ^NS^	0.430 ^NS^	0.062	0.563 ^NS^	0.018 *
**Treatment**	**N**	**P**	**K**	**Ca**	**Mg**	**S**	**Fe**	**Cu**	**Mn**	**Zn**	**B**
	^______________________________^ **(g kg^−1^)** ^________________________________^						^_____________________________^ **(mg kg^−1^)** ^_____________________________^				
T1	24.2 ± 1.9	3.3 ± 0.3	38.1 ± 1.6	1.8 ± 0.2	2.4 ± 0.2	2.0 ± 0.2	73.9 ± 2.1	6.1 ± 0.8	17.0 ± 0.9	17.7 ± 1.5 **b**	13.7 ± 0.6
T2	24.6 ± 1.5	3.3 ± 0.1	35.9 ± 1.6	1.9 ± 0.1	2.4 ± 0.1	2.0 ± 0.1	74.1 ± 8.4	12.0 ± 2.3	17.4 ± 1.3	20.2 ± 1.0 **b**	14.5 ± 0.6
T3	25.2 ± 1.4	3.4 ± 0.3	38.5 ± 2.2	1.8 ± 0.2	2.5 ± 0.2	2.3 ± 0.2	73.0 ± 4.8	7.4 ± 1.0	16.4 ± 0.9	19.9 ± 1.3 **b**	16.2 ± 0.7
T4	26.9 ± 1.8	4.2 ± 0.4	43.1 ± 1.7	2.0 ± 0.2	2.7 ± 0.2	2.3 ± 0.2	86.4 ± 9.4	10.0 ± 1.6	18.9 ± 1.6	29.4 ± 2.8 **a**	15.4 ± 1.9
T5	24.5 ± 1.8	3.2 ± 0.3	37.9 ± 1.2	1.7 ± 0.2	2.4 ± 0.2	2.1 ± 0.1	77.7 ± 4.7	10.2 ± 2.5	16.2 ± 1.1	34.1 ± 3.1 **a**	14.1 ± 0.6
*p*-value	0.772 **^NS^**	0.149 ^NS^	0.102 ^NS^	0.885 ^NS^	0.592 ^NS^	0.495 ^NS^	0.638 ^NS^	0.119 ^NS^	0.592 ^NS^	0.001 *	0.370 ^NS^

T1: Control (without application of multi-nutrient fertilizer (MNF)); T2: Application at the beginning of flowering (2 kg ha^−1^ of MNF providing 5 g of Se ha^−1^); T3: Application at the beginning of flowering +3rd bunch (4 kg ha^−1^ of MNF providing 10 g of Se ha^−1^); T4: Application at the beginning of flowering +6th bunch (4 kg ha^−1^ of MNF providing 10 g of Se ha^−1^); T5: Application at the beginning of flowering +3rd bunch +6th bunch (6 kg ha^−1^ of MNF providing 15 g of Se ha^−1^). **^NS^**: statistically non-significant (*p* > 0.05); *: statistically significant (*p* < 0.05). Means with standard error within each treatment group that shares the same letter in each column are not considered statistically different according to the Duncan test (*p* > 0.05).

**Table 4:** Chemical, physicochemical, and soil particle size distribution of the soil in the study area.

Attributes	Values
pH in water	4.8
Soil organic matter (g kg^−1^)	42.2
Clay (g kg^−1^)	570
Silt (g kg^−1^)	150
Sand (g kg^−1^)	280
Available potassium (mg kg^−1^)	767.31
Available phosphorus (mg kg^−1^)	3271.38
Exchangeable calcium^2+^ (cmol_c_ kg^−1^)	7.73
Exchangeable magnesium^2+^ (cmol_c_ kg^−1^)	1.25
Available zinc (mg kg^−1^)	30
Available iron (mg kg^−1^)	141.2
Available manganese (mg kg^−1^)	46.9
Available copper (mg kg^−1^)	4.37
Available boron (mg kg^−1^)	0.3
Available sulfur (mg kg^−1^)	261.7

Soil pH was determined in water at a ratio of 1:2.5 (*w*/*v*). Soil organic matter was determined by the Walkley–Black method, and clay, silt, and sand were assessed by the Boyoucos method. The available contents of nutrients were determined by the Mehlich-1 soil test. Calcium and magnesium exchangeable contents were extracted by a 1 mol L^−1^ KCl solution–soil test. The available contents of boron were determined by the hot-water extraction method, and the available contents of sulfur were determined by the monocalcium phosphate diluted in acetic acid method. The total content of selenium was also determined. All methodologies are described in Teixeira et al. [49].

**Table 5:** The description of the treatments (Ti) used for the field experiment involving tomato plants.

Treatment	Development Phase of Selenium Application and Rate (kg ha^−1^)
T1	Control (without application of multi-nutrient fertilizer)
T2	Beginning of flowering (2 kg ha^−1^ of multi-nutrient fertilizer providing 5 g of Se ha^−1^)
T3	Beginning of flowering +3rd bunch (4 kg ha^−1^ of multi-nutrient fertilizer providing 10 g of Se ha^−1^)
T4	Beginning of flowering +6th bunch (4 kg ha^−1^ of multi-nutrient fertilizer providing 10 g of Se ha^−1^)
T5	Beginning of flowering +3rd bunch +6th bunch (6 kg ha^−1^ of multi-nutrient fertilizer providing 15 g of Se ha^−1^)

## Data Availability

The data supporting this study’s findings are available on request from the corresponding author.

## Appendix A

**Table A1:** Total content of macro and micronutrients in dried tomato leaf determined according to different strategies of a multi-nutrient fertilizer application containing selenium.

Treatment	N	P	K	Ca	Mg	S	Fe	Cu	Mn	Zn	B
	^______________________________^(g kg^−1^)^________________________________^						^_____________________________^(mg kg^−1^)^_____________________________^				
T1	45.7 ± 0.9	3.5 ± 0.2	47.1 ± 0.9	32.5 ± 1.1	5.2 ± 0.2	17.8 ± 0.7	128.5 ± 5.8	56.2 ± 6.5	101.5 ± 4.7	118.3 ± 11.4	99.5 ± 1.8
T2	47.8 ± 1.6	3.6 ± 0.3	44.9 ± 0.6	33.8 ± 1.3	4.9 ± 0.2	17.9 ± 0.4	134.7 ± 3.0	67.7 ± 6.2	118.5 ± 3.5	134.8 ± 10.1	108.7 ± 3.8
T3	46.9 ± 0.7	3.4 ± 0.2	45.7 ± 1.1	32.0 ± 0.6	5.7 ± 0.2	17.5 ± 0.8	129.8 ± 3.6	58.5 ± 2.5	108.2 ± 6.4	123.0 ± 7.5	102.0 ± 3.9
T4	48.1 ± 0.8	3.7 ± 0.2	44.4 ± 1.3	34.0 ± 0.5	5.2 ± 0.3	17.8 ± 1.1	133.4 ± 5.9	61.7 ± 6.6	111.0 ± 5.0	131.1 ± 8.8	105.9 ± 7.0
T5	48.3 ± 0.8	3.7 ± 0.2	44.4 ± 1.0	34.1 ± 1.2	5.1 ± 0.2	17.2 ± 1.1	136.2 ± 3.1	60.7 ± 4.6	110.3 ± 9.6	125.1 ± 6.4	101.4 ± 4.4
*p*-value	0.479 ^NS^	0.648 ^NS^	0.210 ^NS^	0.461 ^NS^	0.111 ^NS^	0.962 ^NS^	0.656 ^NS^	0.313 ^NS^	0.443 ^NS^	0.550 ^NS^	0.654 ^NS^

T1: Control (without application of multi-nutrient fertilizer); T2: Application at the beginning of flowering (2 kg ha^−1^ of multi-nutrient fertilizer providing 5 g of Se ha^−1^); T3: Application at the beginning of flowering +3rd bunch (4 kg ha^−1^ of multi-nutrient fertilizer providing 10 g of Se ha^−1^); T4: Application at the beginning of flowering +6th bunch (4 kg ha^−1^ of multi-nutrient fertilizer providing 10 g of Se ha^−1^); T5: Application at the beginning of flowering +3rd bunch +6th bunch (6 kg ha^−1^ of multi-nutrient fertilizer providing 15 g of Se ha^−1^). **^NS^**: statistically non-significant (*p* > 0.05).

**Table A2:** Color variables in tomato fruit according to different strategies of multi-nutrient fertilizers’ application containing selenium in each harvest evaluated.

**Tomato Harvest 1 (1st, 2nd, and 3rd bunches)**				
**Treatment**	**L**	**a**	**C**	**h°**
T1	51.89 ± 0.37	15.39 ± 0.64	35.48 ± 0.85	64.40 ± 0.66
T2	52.00 ± 0.63	16.11 ± 0.41	36.19 ± 0.68	63.55 ± 0.60
T3	51.67 ± 0.57	15.92 ± 0.65	35.25 ± 0.20	63.16 ± 1.13
T4	51.27 ± 0.37	15.86 ± 0.45	35.07 ± 0.54	63.52 ± 0.49
T5	51.61 ± 0.72	16.75 ± 0.73	35.78 ± 0.77	62.18 ± 0.94
*p*-value	0.934 **^NS^**	0.715 **^NS^**	0.712 **^NS^**	0.403 **^NS^**
**Tomato Harvest 2 (4th, 5th, and 6th Bunches)**				
**Treatment**	**L**	**a**	**C**	**h°**
T1	51.42 ± 0.74	14.56 ± 0.46	34.46 ± 0.63	65.03 ± 0.81
T2	51.65 ± 0.37	15.92 ± 0.62	36.10 ± 0.30	64.05 ± 1.02
T3	51.08 ± 0.31	16.49 ± 0.95	35.18 ± 0.58	62.17 ± 1.43
T4	51.40 ± 0.34	15.53 ± 0.45	34.59 ± 0.55	63.79 ± 0.45
T5	52.07 ± 0.49	15.93 ± 0.70	35.51 ± 0.94	63.35 ± 0.74
*p*-value	0.531 **^NS^**	0.466 **^NS^**	0.357 **^NS^**	0.312 **^NS^**
**Tomato Harvest 3 (above the 7th Bunches)**				
**Treatment**	**L**	**a**	**C**	**h°**
T1	51.12 ± 0.29	16.02 ± 0.66	35.64 ± 0.35	63.32 ± 0.80
T2	52.20 ± 0.57	17.15 ± 0.77	36.67 ± 0.79	62.37 ± 0.85
T3	51.53 ± 0.49	16.13 ± 0.51	35.40 ± 0.47	63.21 ± 0.96
T4	52.08 ± 0.37	15.87 ± 0.44	35.61 ± 0.70	63.60 ± 0.65
T5	51.46 ± 0.45	16.68 ± 0.20	36.00 ± 0.39	62.36 ± 0.46
*p*-value	0.546 **^NS^**	0.166 **^NS^**	0.260 **^NS^**	0.430 **^NS^**

L: Lightness, a: Red, C: Chroma, h: Hue. T1: Control (without application of multi-nutrient fertilizer); T2: Application at the beginning of flowering (2 kg ha^−1^ of multi-nutrient fertilizer providing 5 g of Se ha^−1^); T3: Application at the beginning of flowering +3rd bunch (4 kg ha^−1^ of multi-nutrient fertilizer providing 10 g of Se ha^−1^); T4: Application at the beginning of flowering +6th bunch (4 kg ha^−1^ of multi-nutrient fertilizer providing 10 g of Se ha^−1^); T5: Application at the beginning of flowering +3rd bunch +6th bunch (6 kg ha^−1^ of multi-nutrient fertilizer providing 15 g of Se ha^−1^). **^NS^**: statistically non-significant (*p* > 0.05).
